# Associations of General and Abdominal Obesity with the Risk of Glioma Development

**DOI:** 10.3390/cancers13122859

**Published:** 2021-06-08

**Authors:** Stephen Ahn, Kyungdo Han, Jung-Eun Lee, Sin-Soo Jeun, Yong-Moon Park, Seung Ho Yang

**Affiliations:** 1 Department of Neurosurgery, Seoul St. Mary’s Hospital, College of Medicine, The Catholic University of Korea, Seoul 06591, Korea; nsstp@catholic.ac.kr (S.A.); ssjeun@catholic.ac.kr (S.-S.J.); 2 Department of Statistics and Actuarial Science, Soongsil University, Seoul 06978, Korea; hkd917@naver.com; 3 Department of Neurosurgery, St. Vincent’s Hospital, College of Medicine, The Catholic University of Korea, Seoul 06591, Korea; eunree@catholic.ac.kr; 4 Department of Epidemiology, Fay W. Boozman College of Public Health, University of Arkansas for Medical Sciences, Little Rock, AR 72205, USA

**Keywords:** waist circumstance, obesity, abdominal obesity, glioma, risk factors

## Abstract

**Simple Summary:**

While obesity is a well-known risk factor for the development of various types of cancer, conflicting results have been reported concerning the relationship between obesity and the risk of glioma. To date, no studies have evaluated the association between obesity and risk of glioma development in Eastern Asian populations, who usually have greater fat mass and less muscle and are more likely to develop several metabolic diseases than Western populations of the same body mass index (BMI) category. In this nationwide population-based study, we suggest, for the first time, positive associations of general and central obesity with the risk of glioma development. In addition, we demonstrate a stronger association between abdominal obesity and the risk of glioma development than BMI and the risk of glioma development.

**Abstract:**

The association between obesity and the risk of glioma remains unclear. We sought to evaluate the potential association between general and abdominal obesity and the risk of glioma based on a nationwide population-based cohort study of Koreans. Using data from the Korean National Health Insurance System cohort, 6,833,744 people older than 20 years who underwent regular national health examination in both 2009 and 2011 were followed until the end of 2017. We documented 4771 glioma cases based on an ICD-10 code of C71 during the median follow-up period of 7.30 years. Individuals with a body mass index (BMI) ≥ 25.0 kg/m^2^ were at significantly higher risk of developing glioma than those with a BMI < 25.0 kg/m^2^ (HR 1.08 CI 1.02–1.15). Individuals with a waist circumference (WC) ≥ 90 cm (males)/85 cm (females) also had a significantly higher risk of glioma than those with a WC < 90 cm (males)/85 cm (females) (HR 1.16 CI 1.09–1.24). In the group with a BMI ≥ 25.0 kg/m^2^, individuals with abdominal obesity were at significantly higher risk of developing glioma (HR 1.18 CI 1.09–1.27) than those without abdominal obesity. The role of abdominal obesity in this association was stronger in women than in men. To the best of our knowledge, this is the first demonstration that obese people may be at higher risk of glioma, especially centrally obese people from an Asian population with a BMI ≥ 25.0 kg/m^2^. Loss of visceral fat in people with abdominal obesity may reduce their risk of developing glioma.

## 1. Introduction

Gliomas refer to a heterogeneous histologic group of tumors, mainly glioblastomas, diffuse astrocytomas, and oligodendrogliomas, and are the most common primary brain malignancy in adults [1,2]. The incidence rate is 6–8 per 100,000 person-years and the prognosis is usually devastating [3]; in the case of glioblastoma, which accounts for more than 70% of all gliomas, the median overall survival is less than 2 years despite multimodal aggressive treatment [4]. Although numerous investigators have sought to identify the risk factors associated with the development of a glioma to inhibit the genesis and progression of these tumors, the etiology of glioma remains unclear [5,6]. In particular, modifiable lifestyle and environmental factors that increase the risk of glioma are poorly understood [5,6].

While obesity is a well-known risk factor for the development of various types of cancer [7,8], conflicting results have been reported concerning the relationship between obesity and the risk of glioma in prospective cohort studies, case–control studies, and meta-analyses [9,10,11,12,13,14,15,16,17,18,19,20,21,22,23]. However, a recent biological study suggested that adipocyte-released factors can promote the growth and progression of glioma cells [24]. Recent clinical studies also supported potential associations between body composition and the progression of tumors in patients with brain malignancies [25,26].

To date, no studies have evaluated the association between obesity and risk of glioma development in Eastern Asian populations, who usually have greater fat mass and less muscle and are more likely to develop several metabolic diseases than Western populations of the same body mass index (BMI) category [27]. BMI, as a surrogate marker for obesity, has been widely used to represent general obesity in numerous studies [7,8]. However, waist circumference (WC) has recently been suggested to be a more sensitive surrogate marker than BMI as WC reflects not only body fat mass but also fat distribution [28,29].

In this context, we sought to evaluate the potential association of obesity with the risk of glioma development in a nationwide population database of Koreans. We adapted WC as a surrogate marker of central body fat mass in addition to BMI as a marker of general obesity in this study.

## 2. Results

### 2.1. Characteristics of the Study Population

Among the 6,833,744 individuals in the study population, a total of 4471 glioma patients were identified during the median follow-up period of 7.30 years. Age, height, weight, waist circumference, BMI, no alcohol consumption, diabetes mellitus, hypertension, and dyslipidemia were significantly associated with the development of a glioma (*p* < 0.001). Detailed baseline characteristics of the study population are provided in Table 1.

### 2.2. Incidence Rates and Risks of Developing Gliomas According to BMI and WC

A total of 4471 gliomas developed during the follow-up period of 49,877,983 person-years. The incidence rate of gliomas per 100,000 person-years was 8.96 in our total study population. In models adjusted for sex, age, and other potential confounding factors, individuals with a BMI ≥ 25.0 kg/m^2^ had a significantly higher risk of developing gliomas than those with a BMI < 25.0 kg/m^2^ (HR 1.08 CI 1.02–1.15 in model 2) (Table 2). When individuals were divided into five groups according to BMI, those with a BMI ≥ 30.0 kg/m^2^ were at higher risk of developing gliomas than those with 18.5 kg/m^2^ ≤ BMI < 23.0 kg/m^2^ (HR 1.27 CI 1.09–1.48 in model 2).

Individuals with a WC ≥ 90 cm (males)/85 cm (females) were at a significantly higher risk of developing gliomas than those with a WC < 90 cm (males)/85 cm (females) (HR 1.16 CI 1.09–1.24 in model 2; Table 2). When individuals were divided into six groups according to WC, males with a WC of 95–99.9 cm and females with a WC of 90–94.9 cm as well as males with a WC ≥ 100 cm and females with a WC ≥ 95 cm were at a significantly higher risk of developing gliomas than males with a WC of 85–89.9 cm and females with a WC of 80–84.9 cm (HR 1.18 CI 1.05–1.34 and HR 1.25 CI 1.07–1.46 in model 2, respectively). When subjects were divided into two groups according to BMI or WC, the association between elevated WC and gliomas was found to be stronger than that between elevated BMI and gliomas (HR 1.16 CI 1.09–1.25 vs. HR 1.08 CI 1.02–1.15).

### 2.3. Impact of Abdominal Obesity on the Association between BMI and Risk of Gliomas

**Figure 1:**
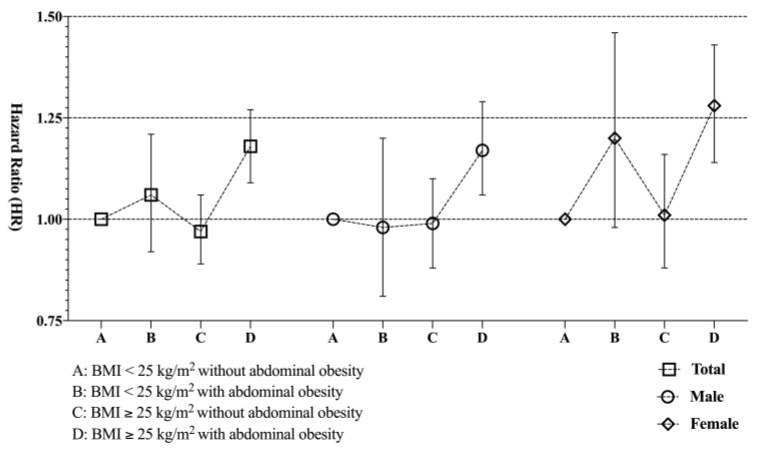
Impact of abdominal obesity on the association between BMI and risk of glioma development.

In subjects with a BMI ≥ 25.0 kg/m^2^, those with abdominal obesity (WC ≥ 90 cm for males and 85 cm for females) had a significantly higher risk of developing gliomas than those individuals in this group without abdominal obesity (HR 1.18 CI 1.09–1.27 in model 2) (Table 3). The impact of abdominal obesity on the associations between BMI and the risk of gliomas in male and female subgroups was similar to that found in the total population (Figure 1).

### 2.4. Subgroup Analysis According to Sex

When we analyzed subgroups according to sex, we found that males with a BMI ≥ 25.0 kg/m^2^ were at a significantly higher risk of glioma development than those with a BMI < 25.0 kg/m^2^ (HR 1.09 CI 1.00–1.18 in model 2) (Table 4). When male subjects were divided into five groups according to BMI, however, there were no significant differences in glioma risk among groups. Females with a BMI ≥ 25.0 kg/m^2^ had a significantly higher risk of glioma development than those with a BMI < 25.0 kg/m^2^ (HR 1.14 CI 1.03–1.25 in model 2) (Table 5). When females were divided into groups according to BMI, females with a BMI ≥ 30.0 kg/m^2^ and females with 25.0 kg/m^2^ ≤ BMI < 30.0 kg/m^2^ were at higher risk of developing gliomas than those with 18.5 kg/m^2^ ≤ BMI < 23.0 kg/m^2^ (HR 1.15 CI 1.03–1.28 and HR 1.42 CI 1.15–1.76 in model 2, respectively).

Males with a WC ≥ 90 cm had a significantly higher risk of glioma development than those with a WC < 90 cm (HR 1.27 CI 1.02–1.49 in model 2) (Table 4). When males were divided into six groups according to WC, males with a WC ≥ 100 cm were at significantly higher risk of developing gliomas than those with 85 cm ≤ WC < 90 cm (HR 1.14 CI 1.04–1.24 in model 2). Females with a WC ≥ 85 cm had a significantly higher risk of developing gliomas than those with a WC < 85 cm (HR 1.26 CI 1.13–1.39 in model 2) (Table 5). When females were divided into six groups according to WC, females with a WC ≥ 95 cm and females with 90 cm ≤ WC < 95 cm were at a significantly higher risk of glioma development than those with 80 cm ≤ WC < 85 cm (HR 1.29 CI 1.04–1.62 and HR 1.27 CI 1.06–1.52 in model 2, respectively). The association between elevated BMI or WC and gliomas was stronger in females than males.

## 3. Discussion

In this study, we demonstrated that individuals with general obesity (BMI ≥ 25.0 kg/m^2^) had a significantly higher risk of developing gliomas than those without general obesity (BMI < 25.0 kg/m^2^). We also found that individuals with abdominal obesity (WC ≥ 90 cm for males/85 cm for females) were at significantly higher risk of developing gliomas than those without abdominal obesity. This association of general obesity and abdominal obesity with the risk of glioma development was stronger in women than men. Interestingly, subgroup analysis showed that individuals with both general obesity and abdominal obesity were at significantly higher risk of developing gliomas, while those with general obesity but without abdominal obesity exhibited no increase in risk of glioma development compared to those individuals with a normal BMI. To the best of our knowledge, this is the first study to suggest positive associations of general and central obesity with the risk of glioma development. In addition, we found a stronger association between abdominal obesity and the risk of glioma development than BMI and the risk of glioma development. Using a nationwide population database collected from the NHIS of Koreans, our study included a large number of glioma cases (*n* = 4771) and is the second largest study to evaluate the association between obesity and glioma risk [9]. In addition, our findings about well-known risk factors such as sex and age were in accordance with the latest reported study; the adjusted HR of age increase per year is 1.06 (CI 1.05–1.06), and that of female sex is 0.84 (CI 0.78–0.91) compared to males.

Numerous epidemiological studies have shown associations between excess body fat and increased risk of developing various types of cancer, including thyroid cancer, esophageal adenocarcinoma, and cancers of the stomach, pancreas, colorectum, gallbladder, breast, endometrium, and kidney, although there are variations between sexes and among different ethnic populations [27,30,31,32]. Recent studies have described potential pathophysiological mechanisms that link obesity to cancer [33,34]. An increase in the amount of adipose tissue, which functions as an endocrine organ, can lead to increased levels of hormones that can play a role in carcinogenesis, such as insulin-like growth factor (IGF) [33,34]. Hyperadiposity is positively associated with insulin resistance, which induces hyperinsulinemia [35]. Excess insulin in turn decreases the hepatic secretion of IGF-binding proteins, which results in increased circulating IGF-1 [36]. IGF-1 induces angiogenesis via the synthesis of hypoxia inducible factor-1a (HIF-1a) [36]. Furthermore, adiponectin, as a critical protein secreted from adipocytes, is an important regulator of insulin sensitivity and fat metabolism [37]. Alterations in circulating levels of these hormones can inhibit apoptosis and promote neo-vascularization, which contributes to the initiation and progression of cancer [34,35]. In addition, hyperadiposity is associated with higher levels of circulating inflammatory markers, including adiponectin, interleukin (IL)-6, IL-1b, tumor necrosis factor-a, and cylocoxygenase-2. These inflammatory proteins induce chronic inflammation of adipose tissue and alter the microenvironment to favor tumor initiation and progression [33,38]. Hyperadiposity can both initiate and sustain a tumor-promoting microenvironment through systemic and local pathways [38].

Several cohort studies, case–control studies, and meta-analyses have evaluated the associations between adiposity and gliomas; however, the results from these studies have been inconclusive. While the majority of these studies have reported null associations [9,10,11,12,13,14,15,16,17,18,19,20,21,22,23], three studies reported positive associations [14,18,22]; one meta-analysis study found that there was a significant association between adiposity and glioma development in females [14], while one cohort study and one case–control study found that elevated BMI in early adulthood was significantly associated with increased risk of gliomas [18,22]. These previous studies had some limitations. First, although elevated BMI is a major risk factor for the development of metabolic disorders such as hypertension, diabetes, high cholesterol, and central obesity, these metabolic abnormalities could also affect the risk of developing cancer independently, even in normal-weight individuals [39]. Among previous studies that have evaluated the associations between obesity and glioma development, none have performed subgroup analyses according to metabolic abnormalities in different BMI groups. Combinational or synergistic effects of general obesity and metabolic abnormalities were also not evaluated in these previous studies. Second, these studies included only individuals from Western populations. East Asian populations have a 3–5% higher body fat mass for the same BMI compared to Western populations [27,40] and are at higher risk of developing metabolic diseases at the same or a lower BMI than their Western counterparts [40]. Lastly, there can be weight loss from the time of gliomagenesis to diagnosis in glioma patients, and weight was measured at the time of diagnosis in one of these previous studies [41]. This may have resulted in an underestimation of the association between obesity and risk of glioma development.

Given this background, our goal was to validate potential associations between obesity and the risk of glioma development in a nationwide population database of Koreans. To minimize bias due to the limitations of BMI as an indirect measure of body fat mass, we also evaluated WC, as this has been suggested to be a more sensitive marker of central body fat mass. Our results suggest that obese Asians have a higher risk of developing gliomas, especially centrally obese individuals with a BMI ≥ 25 kg/m^2^. Our findings are supported by a recent biological study that demonstrated that adipocyte-released factors induce the growth and progression of glioma cells [24]. Recent clinical studies that have shown that body composition is related to brain tumor progression, including glioblastoma multiforme progression, are also consistent with our findings [25,26]. Taken together, these results suggest that physicians should consider recommending that patients with general and abdominal obesity take measures to reduce their visceral fat mass in order to reduce their risk of developing a glioma.

This study had several limitations that should be considered. First, gliomas comprise heterogeneous subtypes, which can show different levels of susceptibility to obesity. However, our study did not include pathologic findings to define glioma subtypes. Further studies are needed to analyze incidence and risk factors for histologic and molecular subgroups of gliomas. Second, exact body fat mass was not measured; rather, we used WC as a surrogate marker of obesity. Third, although we included many potential risk factors for gliomas, there could be several undefined factors that were not included. Fourth, although we validated the accuracy of our methodology to identify gliomas by retrospectively reviewing electronic medical records at a tertiary referral hospital in Korea, these results may not be generalizable to other hospitals in Korea. Fifth, although obesity is a well-known risk factor in various cancer types, the previous studies on obesity and glioma incidence showed controversial or null results. Further larger and prospective studies are needed to validate our findings.

## 4. Materials and Methods

### 4.1. Ethical Considerations

This study was performed in accordance with the ethical standards of the 1964 Declaration of Helsinki. The Institutional Review Board of Seoul St. Mary’s Hospital approved the study design (ethical code: KC18ZESI0648, permission date: 23 October 2018). To protect individuals’ information, all data were anonymized. Due to the retrospective manner of the study, the requirement for informed consent was waived.

### 4.2. Database Resource

This retrospective nationwide population-based study was conducted using the database of the National Health Insurance Service (NHIS) of Korea, which is a mandatory health insurance system operated by the Korean government that covers almost all Koreans (97%, approximately 50 million people) [28,42,43]. The NHIS database includes demographic information as well as medical information such as clinical diagnoses, prescribed medications including chemotherapy, surgical procedures, and radiotherapy. In addition, this database contains physical measurements, including height, weight, BMI, medical history, family history, and socio-behavioral history including cigarette smoking, alcohol consumption, and physical activity, which are obtained from self-reported questionnaire records through a regular national health examination that is provided by the NHIS, either for all enrolled adults >40 years old at least every two years, or for any workers at a company >20 years old.

### 4.3. Study Population

**Figure 2:**
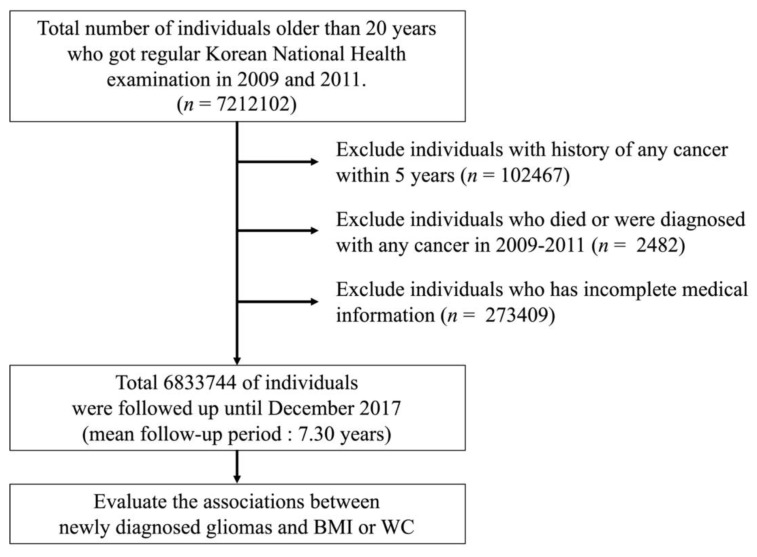
Flow of enrollment of study participants.

We reviewed records from the NHIS database for all people who were older than 20 years by 2009. Because the NHIS limits its database size to 10 million people due to the personal information protection policy, 6k833,744 people were identified after including people who underwent a regular national health examination annually or biennially both in 2009 and 2011 and excluding people with any cancer history and/or incomplete medical information. The overall flow of patient enrollment is illustrated in Figure 2. In this population, a total of 4471 gliomas were identified from January 2009 to December 2017. The mean follow-up periods for enrolled individuals were 7.30 years and 49,877,983 person-years.

### 4.4. Definition of Glioma

The medical code “C71” represents intracranial gliomas according to the International Classification of Disease, Tenth Revision (ICD-10), and includes diffuse astrocytomas, anaplastic astrocytomas, ependymomas, anaplastic ependymomas, oligodendrogliomas, anaplastic oligodendrogliomas, and glioblastoma multiforme. Because all C71 patients received an additional cost coverage service from the NHIS for rare and incurable diseases, called the “benefit extension policy for rare incurable diseases (BEP)”, we defined patients with gliomas as those who were diagnosed with C71 and who were registered as having a BEP to ensure more accurate identification of glioma patients [42]. To verify the accuracy of our method of identifying gliomas, we retrospectively reviewed the electronic medical records at Seoul St. Mary’s Hospital, a tertiary referral hospital in Korea. After evaluating medical records for patients who fit our definition of diagnosis of a glioma between 2014 and 2018, we confirmed that, among a total of 220 patients, all had been diagnosed with glioma pathologically or radiologically [42].

### 4.5. Clinical Variables

WC was measured at the midlevel between the lower ribs and iliac crest using a tape measure in a standing position by healthcare providers. Abdominal obesity was defined as a WC ≥ 90 cm for men and ≥85 cm for women based on the World Health Organization’s recommendations for Asians [44]. BMI was calculated as weight (kg) divided by the square of height in meters (m^2^). Individuals were categorized into five groups according to BMI based on the World Health Organization’s recommendations for Asians [44]: underweight (<18.5 kg/m^2^); normal (18.5–22.9 kg/m^2^); overweight (23.0–24.9 kg/m^2^); obese class I (25.0–29.9 kg/m^2^); and obese class II (>30.0 kg/m^2^).

Current smokers were defined as people who smoked more than five packs (a total of 100 cigarettes) over their lifetime and continued to smoke; former smokers were defined as people who had smoked more than five packs (a total of 100 cigarettes) over their lifetime, but who quit smoking prior to completing the questionnaire; and never-smokers were defined as people who had smoked five packs or fewer over their lifetime. Heavy drinkers were defined as individuals who consumed an average of more than 30 g of alcohol per day and mild drinkers were defined as individuals who consumed an average of less than 30 g of alcohol per day. Regular exercise was defined as intensive physical activity with faster-than-normal breathing for ≥ 20 min at a time more than 3 days per week, and moderate physical activity was defined as slightly faster-than-normal breathing ≥30 min at least 5 days per week. Diabetes mellitus was defined as a fasting plasma glucose level ≥126 mg/dL or use of insulin or oral hypoglycemic agents. Hypertension was defined as a systolic blood pressure ≥ 140 mmHg and/or a diastolic blood pressure ≥90 mmHg or use of antihypertensive agents. Dyslipidemia was defined as a total cholesterol level ≥240 mg/dL or use of lipid-lowering agents.

### 4.6. Statistical Analyses

Data are expressed as mean ± standard deviation for continuous variables and as proportions for categorical variables. One-way analysis of variance was used to compare differences between continuous variables, and the chi-square test was used to compare differences between categorical variables. Incidence rates for gliomas were calculated and expressed as the number of events per 100,000 person-years. We fit a model adjusted for the potential confounders of age and sex as model 1, and we fit another model that included the potential confounders from model 1 in addition to smoking status, alcohol consumption, exercise level, and the presence of diabetes mellitus (model 2). Cumulative incidence rates for gliomas were compared between groups using the Kaplan–Meier method and the log-rank test. Cox proportional hazards models were used to analyze the adjusted risk of developing gliomas, based on BMI and WC; results are expressed as hazard ratios (HR) with 95% confidence intervals (CI). A *p*-value < 0.05 was considered statistically significant. Statistical analyses were performed using SAS version 9.4 (SAS Institute, Cary, NC, USA).

## 5. Conclusions

This nationwide cohort study firstly suggests that obese people may be at higher risk of developing gliomas, especially centrally obese people from an Asian population with a BMI ≥ 25.0 kg/m^2^. Loss of visceral fat in people with abdominal obesity may reduce their risk of developing gliomas.

## Figures and Tables

**Table 1:** Baseline characteristics of the study population.

*n* (%)	Healthy Individuals	Glioma Patients	*p* Value
	*n* = 6,829,273	*n* = 4471	
Mean age, years ^a^	46.84 ± 13.53	56.65 ± 13.01	<0.001
Male	3,923,066 (57.44)	2553 (57.1)	0.642
Height, cm ^a^	164.35 ± 9.21	162.46 ± 9.14	<0.001
Weight, kg ^a^	64.42 ± 11.53	63.81 ± 10.93	0.004
Waist circumference, cm ^a^	80.4 ± 9.3	82.5 ± 8.4	<0.001
<80 ^b^/<75 ^c^	2,471,470 (36.19)	1193 (26.68)	
80–84.9 ^b^/75–79.9 ^c^	1,667,249 (24.41)	1024 (22.90)	
85–89.9 ^b^/80–84.9 ^c^	1,378,370 (20.18)	1036 (23.17)	
90–94.9 ^b^/85–89.9 ^c^	790,962 (11.58)	679 (15.19)	
95–99.9 ^b^/90–94.9 ^c^	342,326 (5.01)	349 (7.81)	
≥100 ^b^/≥95 ^c^	178,896 (2.62)	190 (4.25)	
BMI, kg/m ^a^	23.76 ± 3.48	24.1 ± 3.14	<0.001
<18.5	223,786 (3.28)	99 (2.21)	
18.5–22.9	2,622,565 (38.4)	1554 (34.76)	
23.0–24.9	1,729,528 (25.33)	1154 (25.81)	
25.0–29.9	2,022,217 (29.61)	1483 (33.17)	
≥30.0	231,177 (3.39)	181 (4.05)	
Smoker			0.003
None	3,978,206 (58.25)	2659 (59.47)	
Former	1,053,665 (15.43)	731 (16.35)	
Current	1,797,402 (26.32)	1081 (24.18)	
Drinker			<0.001
None	3,412,478 (49.97)	2631 (58.85)	
Mild	2,874,998 (42.1)	1502 (33.59)	
Heavy	541,797 (7.93)	338 (7.56)	
Regular exercise	1,282,475 (18.78)	895 (20.02)	0.034
Diabetes mellitus	558,664 (8.18)	611 (13.67)	<0.001
Hypertension	959,082 (14.04)	912 (20.4)	<0.001
Systolic BP ^a^	122.4 ± 14.7	125.51 ± 15.3	
Diastolic BP ^a^	76.4 ± 9.9	77.4 ± 9.9	
Dyslipidemia	1,233,872 (18.07)	1095 (24.49)	<0.001
Total cholesterol	195.6 ± 40.7	197.7 ± 43.1	

BMI, body mass index; BP, blood pressure; HDL, high-density lipoprotein; LDL, low-density lipoprotein; *n*, number. ^a^ Described as mean ± standard deviation; ^b^ for males; ^c^ for females.

**Table 2:** Incidence rates and risk of glioma development according to BMI and WC.

	Total, *n*	Glioma Events, *n*	Person-Years	Incidence Rates *	Crude (95% CI)	Model 1 HR ^†^ (95% CI)	Model 2 HR ^‡^ (95% CI)
BMI (kg/m^2^)							
<25.0	4,578,686	2807	33,403,700	8.40	1 (reference)	1 (reference)	1 (reference)
≥25.0	2,255,058	1664	16,474,282	10.10	1.20 (1.13–1.28)	1.08 (1.02–1.15)	1.08 (1.02–1.15)
BMI (kg/m^2^)							
<18.5	223,885	99	1,616,699	6.12	0.76 (0.62–0.93)	0.93(0.76–1.14)	0.915(0.75,1.12)
18.5–22.9	2,624,119	1554	19,139,370	8.12	1 (reference)	1 (reference)	1 (reference)
23.0–24.9	1,730,682	1154	12,647,631	9.12	1.12 (1.04–1.21)	0.97 (0.90–1.05)	0.97 (0.90–1.05)
25.0–29.9	2,023,700	1483	14,786,709	10.03	1.24 (1.15–1.33)	1.04 (0.97–1.12)	1.04 (0.97–1.12)
≥30.0	231,358	181	1,687,572	10.73	1.32 (1.13–1.54)	1.29 (1.10–1.50)	1.27 (1.09–1.48)
WC (cm)							
<90 ^a^/85 ^b^	5,520,342	3253	40,319,911	8.07	1 (reference)	1 (reference)	1 (reference)
≥90 ^a^/85 ^b^	1,313,402	1218	9,558,071	12.74	1.58 (1.48–1.69)	1.17 (1.09–1.25)	1.16 (1.09–1.24)
WC (cm)							
<80 ^a^/<75 ^b^	2,472,663	1193	18,068,195	6.60	0.64 (0.59–0.70)	0.95 (0.87–1.03)	0.95 (0.87–1.03)
80–84.9 ^a^/75–79.9 ^b^	1,668,273	1024	12,185,415	8.40	0.82 (0.75–0.89)	0.93 (0.85–1.01)	0.93 (0.85–1.01)
85–89.9 ^a^/80–84.9 ^b^	1,379,406	1036	10,066,300	10.23	1 (reference)	1 (reference)	1 (reference)
90–94.9 ^a^/85–89.9 ^b^	791,641	679	5,766,604	11.78	1.14 (1.04–1.26)	1.05 (0.96–1.16)	1.05 (0.95–1.16)
95–99.9 ^a^/90–94.9 ^b^	342,675	349	2,492,630	14.00	1.36 (1.20–1.54)	1.19 (1.05–1.34)	1.18 (1.05–1.34)
≥100 ^a^/≥95 ^b^	179,086	190	1,298,836	14.63	1.42 (1.22–1.66)	1.27 (1.09–1.49)	1.25 (1.07–1.46)

BMI, body mass index; CI, confidence interval; HR, hazard ratio; *n*, number; WC, waist circumference; * per 100,000 person-years; ^†^ Model 1: adjusted for age and sex; ^‡^ Model 2: adjusted for model 1 plus smoking status, alcohol consumption, exercise level, and the presence of diabetes mellitus; ^a^ for males; ^b^ for females.

**Table 3:** Impact of abdominal obesity on the association between BMI and risk of glioma development.

	BMI (kg/m^2^)	WC (cm)	Total, *n*	Glioma Events, *n*	Person-Years	Incidence Rates *	Crude (95% CI)	Model 1 HR ^†^ (95% CI)	Model 2 HR ^‡^ (95% CI)
Total									
	<25.0	<90 ^a^/85 ^b^	4,374,943	2588	31,934,209	8.10	1 (reference)	1 (reference)	1 (reference)
		≥90 ^a^/85 ^b^	203,743	219	1,469,491	14.90	1.84 (1.60–2.11)	1.06 (0.92–1.22)	1.06 (0.92–1.21)
	≥25.0	<90 ^a^/85 ^b^	1,145,399	665	8,385,701	7.93	0.98 (0.89–1.07)	0.97 (0.89–1.05)	0.97 (0.89–1.06)
		≥90 ^a^/85 ^b^	1,109,659	999	8,088,580	12.35	1.52 (1.42–1.64)	1.18 (1.10–1.27)	1.18 (1.09–1.27)
Males									
	<25.0	<90 ^a^/85 ^b^	2,353,787	1480	17,108,111	8.65	1 (reference)	1 (reference)	1 (reference)
		≥90 ^a^/85 ^b^	107,077	110	764,599	14.39	1.66 (1.37–2.02)	0.99 (0.81–1.20)	0.98 (0.81–1.20)
	≥25.0	<90 ^a^/85 ^b^	758,916	402	5,543,757	7.25	0.84 (0.75–0.94)	0.97 (0.87–1.08)	0.99 (0.88–1.10)
		≥90 ^a^/85 ^b^	705,839	561	5,124,786	10.94	1.27 (1.15–1.40)	1.16 (1.05–1.28)	1.17 (1.06–1.29)
Females									
	<25.0	<90 ^a^/85 ^b^	2,021,156	1108	14,826,098	7.47	1 (reference)	1 (reference)	1 (reference)
		≥90 ^a^/85 ^b^	96,666	109	704,891	15.47	2.07 (1.70–2.52)	1.20 (0.98–1.47)	1.20 (0.98–1.46)
	≥25.0	<90 ^a^/85 ^b^	386,483	263	2,841,943	9.25	1.24 (1.08–1.42)	1.02 (0.89–1.16)	1.01 (0.88–1.16)
		≥90 ^a^/85 ^b^	403,820	438	2,963,794	14.78	1.98 (1.77–2.21)	1.29 (1.15-1.45)	1.28 (1.14–1.43)

BMI, body mass index; CI, confidence interval; HR, hazard ratio; *n*, number; WC, waist circumference; * per 100,000 person-years; ^†^ Model 1: adjusted for age and sex; ^‡^ Model 2: adjusted for model 1 plus smoking status, alcohol consumption, exercise level, and the presence of diabetes mellitus; ^a^ for males; ^b^ for females.

**Table 4:** Incidence rates and risks of glioma development according to BMI and WC in males.

	Total, *n*	Glioma Events, *n*	Person-Years	Incidence Rates *	Crude (95% CI)	Model 1 HR ^†^ (95% CI)	Model 2 HR ^‡^ (95% CI)
BMI (kg/m^2^)							
<25.0	2,460,864	1590	17,872,711	8.90	1 (reference)	1 (reference)	1 (reference)
≥25.0	1,464,755	963	10,668,544	9.03	1.02 (0.94–1.10)	1.07 (0.99–1.16)	1.09 (1.00–1.18)
BMI (kg/m^2^)							
<18.5	77,240	51	546,617	9.33	1.02 (0.77–1.35)	0.92 (0.69–1.22)	0.89 (0.67–1.18)
18.5–22.9	1,304,404	872	9,464,095	9.21	1 (reference)	1 (reference)	1 (reference)
23.0–24.9	1,079,220	667	7,861,998	8.48	0.92 (0.83–1.02)	0.90 (0.814, 0.996)	0.92 (0.83–1.02)
25.0–29.9	1,327,881	879	9,673,680	9.09	0.99 (0.90–1.08)	1.00 (0.91–1.10)	1.03 (0.93–1.13)
≥30.0	136,874	84	994,863	8.44	0.92 (0.73–1.15)	1.23 (0.99–1.54)	1.24 (0.99–1.56)
WC (cm)							
<90	3,112,703	1882	22,651,869	8.31	1 (reference)	1 (reference)	1 (reference)
≥90	812,916	671	5,889,385	11.39	1.37 (1.26–1.50)	1.13 (1.04–1.24)	1.14 (1.04–1.24)
WC (cm)							
<80	1,153,492	611	8,393,298	7.28	0.74 (0.66–0.82)	0.93 (0.83–1.04)	0.91 (0.82–1.02)
80–84.9	1,071,622	632	7,804,377	8.10	0.82 (0.73–0.91)	0.90 (0.81–1.01)	0.90 (0.80–1.01)
85–89.9	887,589	639	6,454,193	9.90	1 (reference)	1 (reference)	1 (reference)
90-94.9	510,969	395	3,706,459	10.66	1.07 (0.95–1.22)	1.00 (0.88–1.13)	1.00 (0.88–1.13)
95–99.9	204,118	184	1,477,318	12.46	1.26 (1.07–1.48)	1.15 (0.97–1.35)	1.14 (0.97–1.35)
≥100	97,829	92	705,608	13.04	1.32 (1.06–1.64)	1.29 (1.04–1.60)	1.27 (1.02–1.59)

BMI, body mass index; CI, confidence interval; HR, hazard ratio; *n*, number; WC, waist circumference; * per 100,000 person-years; ^†^ Model 1: adjusted for age and sex; ^‡^ Model 2: adjusted for model 1 plus smoking status, alcohol consumption, exercise level, and the presence of diabetes mellitus.

**Table 5:** Incidence rates and risks of glioma development according to BMI and WC in females.

	Total, *n*	Glioma Events, *n*	Person-Years	Incidence Rates *	Crude (95% CI)	Model 1 HR ^†^ (95% CI)	Model 2 HR ^‡^ (95% CI)
BMI (kg/m^2^)							
<25.0	2,117,822	1217	15,530,989	7.84	1 (reference)	1 (reference)	1 (reference)
≥25.0	790,303	701	5,805,738	12.07	1.54 (1.40–1.69)	1.15 (1.04–1.26)	1.14 (1.03–1.25)
BMI (kg/m^2^)							
<18.5	146,645	48	1,070,081	4.49	0.64 (0.48–0.85)	0.90 (0.67–1.20)	0.90 (0.67–1.20)
18.5–22.9	1,319,715	682	9,675,274	7.05	1 (reference)	1 (reference)	1 (reference)
23.0–24.9	651,462	487	4,785,632	10.18	1.44 (1.29–1.62)	1.11 (0.98–1.24)	1.10 (0.98–1.24)
25.0–29.9	695,819	604	5,113,028	11.81	1.68 (1.50–1.87)	1.16 (1.03–1.29)	1.15 (1.03–1.28)
≥30.0	94,484	97	692,709	14.00	1.99 (1.60–2.46)	1.45 (1.17–1.80)	1.42 (1.15–1.76)
WC (cm)							
<85	2,407,639	1371	17,668,041	7.76	1 (reference)	1 (reference)	1 (reference)
≥85	500,486	547	3,668,685	14.91	1.92 (1.74–2.12)	1.27 (1.14–1.41)	1.26 (1.13–1.39)
WC (cm)							
<75	1,319,171	582	9,674,896	6.02	0.54 (0.48–0.62)	0.91 (0.80–1.04)	0.92 (0.80–1.05)
75–79.9	596,651	392	4,381,038	8.95	0.81 (0.71–0.94)	0.95 (0.83–1.10)	0.96 (0.83–1.10)
80–84.9	491,817	397	3,612,106	11.00	1 (reference)	1 (reference)	1 (reference)
85–89.9	280,672	284	2,060,145	13.76	1.26 (1.08–1.46)	1.15 (0.98–1.34)	1.14 (0.98–1.33)
90–94.9	138,557	165	1,015,312	16.25	1.48 (1.23–1.77)	1.27 (1.06–1.53)	1.27 (1.06–1.52)
≥95	81,257	98	593,228	16.52	1.50 (1.21–1.88)	1.31 (1.05–1.63)	1.29 (1.04–1.62)

BMI, body mass index; CI, confidence interval; HR, hazard ratio; *n*, number; WC, waist circumference; * per 100,000 person-years; ^†^ Model 1: adjusted for age and sex; ^‡^ Model 2: adjusted for model 1 plus smoking status, alcohol consumption, exercise level, and the presence of diabetes mellitus.

## Data Availability

Data available on request due to privacy/ethical restrictions.
